# An Assessment of Current and Past Concentrations of Trihalomethanes in Drinking Water throughout France

**DOI:** 10.3390/ijerph15081669

**Published:** 2018-08-06

**Authors:** Magali Corso, Catherine Galey, René Seux, Pascal Beaudeau

**Affiliations:** 1 French National Public Health Agency, 94415 Saint-Maurice, France; catherine.galey@santepubliquefrance.fr (C.G.); pascal.beaudeau@santepubliquefrance.fr (P.B.); 2 French School of Public Health (EHESP), 35043 Rennes, France; rene.seux0637@orange.fr

**Keywords:** chlorination by-product, France, environmental exposure, organic matter, tap water, trihalomethanes

## Abstract

In France, 95% of people are supplied with chlorinated tap water. Due to the presence of natural organic matter that reacts with chlorine, the concentrations of chlorination by-products (CBPs) are much higher in chlorinated water produced from surface water than from groundwater. Surface water supplies 33% of the French population. Until the 1980s, almost all surface water utilities pre-chlorinated water at the intake. Pre-chlorination was then gradually banned from 1980 to 2000. Trihalomethanes (THMs) are the only regulated CBP in France. Since 2003, THMs have been monitored at the outlet of all utilities. This study assessed current (2005–2011) and past (1960–2000) exposure of the French population to THMs. We developed an original method to model THM concentrations between 1960 and 2000 according to current concentrations of THMs, concentration of total organic carbon in raw and finished water, and the evolution of water treatments from 1960 onward. Current and past mean exposure of the French population to THMs was estimated at 11.7 µg·L^−1^ and 17.3 µg·L^−1^, respectively. In the past, approximately 10% of the French population was exposed to concentrations >50 µg·L^−1^ vs. 1% currently. Large variations in exposure were observed among France’s 100 administrative districts, mainly depending on the water origin (i.e., surface vs. ground), ranging between 0.2 and 122.1 µg·L^−1^ versus between 1.8 and 38.6 µg·L^−1^ currently.

## 1. Introduction

Today, almost all the French population is supplied with chlorinated tap water. Chlorination can interact with organic matter dissolved in the water, leading to the formation of unwanted and potentially toxic chlorination by-products (CBPs). The first CBPs were identified in drinking water in the early 1970s with the detection of chloroform and other organohalides [1]. As analytical methods improved, the number of CBPs identified in tap water increased considerably and now includes over 750 substances [2]. The toxicity of several of these substances has been evaluated, and some, including chloroform, bromodichloromethane, and dichloroacetic acid, are considered possible carcinogens for humans (group 2B) by the International Agency for Research on Cancer (IARC) [3].

In France, approximately 33% of the population is supplied by large drinking water treatment plants (DWTPs) fed by surface water, and 67% by smaller DWTPs fed by groundwater [4,5]. Because of its higher organic matter, surface water has a greater potential for CBP formation than groundwater. Some karstic and alluvial groundwater bodies influenced by surface water may also contain a non-negligible amounts of organic matter. The CBP formation potential depends mainly on the effectiveness of the treatment process used to eliminate organic matter, as well as on the chlorination process. Prechlorination of surface water—banned in France since 2000—required higher doses of chorine than chlorination at the outlet of the DWTP and was therefore responsible for the development of many more CBPs.

The population’s main source of exposure to CBP is tap water, through ingestion, inhalation, and skin absorption [6]. The latter two are responsible for the greatest exposure to most CBPs, especially trihalomethanes (THMs), the exposure to which mainly occurs during showers and baths [7]. THMs are the most common CBPs, and the first to have been studied and regulated. The regulatory limit has been set at 100 μg·L^−1^ in France since 2008, while in the U.S. and Quebec, it is 80 μg·L^−1^. In Europe, they are the only regulated CBP. THM analytical data are widely used as indicators of exposure to CBPs in tap water. Many studies have shown a link between THM concentrations in tap water and bladder cancer in men, a link confirmed by meta-analyses [8,9] and pooled analyses [9,10] of the most robust studies. The average THM concentration, as measured at the consumer’s tap or at the outlet of DWTP, is the indicator most often used in epidemiological studies [11,12,13,14]. Using the data from a Spanish case-control study, Salas showed that average concentration over forty years was the most appropriate THM-based indicator for studying the risk of bladder cancer [15].

We used the updated exposure-risk function published in 2011 by Costet et al. [9] to conduct a quantitative health impact assessment of CBP in water intended for human consumption in France [16]. This paper describes the levels of THMs and their trend in French water distribution systems between 1960 and 2011, and the use of water monitoring data for past (1960–2000) exposure assessment.

## 2. Materials and Methods

The geographical unit we used to determine the population’s exposure to CBP was the water distribution zone (WDZ). WDZs are fed by one or more DWTP. The water quality data used come from samples collected from DWTP water.

Since THM data were not available before 2000, past concentrations were modelled. For this purpose, we developed methods that account for changes that occurred in treatment practices from 1960 onwards for both ground and surface water.

After estimating mean concentrations for 1960–2000 and 2005–2011 for all WDZs throughout France, we calculated administrative district (called département in France) and national averages, by weighting WDZ values by the size of the population supplied.

### 2.1. Water Data

DWTP data (location, type of water used, treatment facilities, towns and populations served) and water analytical data were extracted from the national database SISE-Eaux, which is used to monitor tap-water quality. The THM concentration data used in this study came from samples taken at the outlet of all DWTPs under regulatory monitoring from 2005 to 2011. From the SISE-Eaux database, we extracted 88,350 THM analyses from 13,732 DWTPs. Analysis of THMs was performed with a gas chromatography and mass spectrometry system by a static headspace technique (HS-GC-MS) according to the ISO 20595 standard [17]; or with a Purge and Trap and gas chromatography system according to the ISO 15680 standard [18] (Water quality—Determination of selected highly volatile organic compounds in water—Method using gas chromatography and mass spectrometry by static headspace technique (HS-GC-MS)) adapted to THM concentration measurement in tap water following the ISO 5725 standard [19].

The drinking water produced in France from surface water has always undergone post-chlorination. That is not the case for groundwater, a significant proportion being distributed without chlorination before 2003. In 2003, health authorities promoted post-chlorination [20] of groundwater and it was gradually implemented into all of France’s DWTPs.

To identify when water chlorination of a given groundwater DWTP commenced, we looked for the earliest chlorine-free analyses in SISE-Eaux. Since no chlorination data were available before 2000, we assumed that DWTPs with no chlorine analysis after 2000 were not equipped with chlorination facilities before 2000. When data were available for the period 2000–2003, we assumed that, for the 1960–2000 period, the serviced population was supplied with chlorinated water. Approximately 130,000 samples of free chlorine were collected in 9400 DWTP supplied with groundwater between 2000 and 2003, i.e., 66% of these DWTP and 90% of the total population supplied with groundwater.

Our method to model the past concentrations of THMs (see section “Estimating past average concentrations of THM”) also necessitated having total organic carbon (TOC) data. A total of 33,496 TOC values taken from analyses of raw water, and 68,604 from analyses of treated water, were extracted from the SISE-Eaux database.

### 2.2. Processing of the THM Measurements below the Limit of Quantification

Seventy-seven percent of the THM analyses had at least one compound whose concentration was below the limit of quantification (LQ). For 37% of the THM analyses, all the parameters measured were below the LQ. We therefore produced two estimates of average concentration per DWTP, a high and a low estimate calculated by assigning to the values below the LQ the LQ value or zero, respectively. The mean estimate, as referred in the “Results section”, is the average of the low and high estimates.

### 2.3. Correction for the Seasonality of THM Concentrations

The combination of seasonal effects of both sampling frequency and THM concentrations resulted in potential bias in the assessment of THM average concentrations that we analyzed and corrected. As the seasonal effect on THM concentration varies depending on local climate, we firstly modeled by a spline function the seasonal variations at the district level, using available THM data for all DWTPs fed by surface water in the district. We then weighted the THM measurements made for each DWTP.

### 2.4. Estimating the Current Average Concentrations of THM 

The current exposure of the population living in a given WDZ was defined as the average THM concentration of water for each DWTP feeding this WDZ for the 2005–2011 period. Data were corrected for seasonal variations affecting both THM concentrations and sampling plans. The latter were developed at the district level (i.e., smaller than the WDZ level) and possibly did not target same seasons. Accordingly, to correct for this, we calculated the average seasonal component of THM concentrations at the district level and referred to this distribution to correct the available data sample at the WDZ scale. Average concentrations of THM in the WDZs were estimated as the average of all selected concentration measurements.

#### 2.4.1. Estimating Past Average Concentrations of THM in Treated Surface Water

Two different methods were used to estimate THM levels in treated surface water. Both take into account the greater exposure to THM at the outlet of DWTP caused by prechlorination (vs. postchlorination) prior to 2000. We assumed that all DWTP ran prechlorination before 1960 and gradually stopping it from 1980 onwards, before it was finally banned in 2000.

The first method (Method 1) was used by Chevrier et al. in their case-control study [12]. It uses the results of a pilot experiment reproducing types of treatment facilities in use before 2000, and focuses on the impact of different chlorination practices on THM formation. Average concentrations of THM ranging from 0 to 78 µg·L^−1^ were found for the eight (i.e., four each for ground and surface water) most common combinations of treatments. Two of these combinations prevailed in DWTP using surface water: (i) treatment including both pre-chlorination and post-chlorination and (ii) post-chlorination but no pre-chlorination. The average THM concentration was estimated, respectively, at 78.3 µg·L^−1^ and 31.8 µg·L^−1^ (i.e., 2.5 times lower).

We chose this ratio (2.5) of THM concentrations (THM_2005–2011_) to estimate past values THM_pre-chlo_ (i.e., before pre-chlorination was banned) from the current value THM_2005–2011_.

THM*_pre_chlo_* = THM_2005–2011_ × 2.5 (1)

The second method (Method 2) is an original method which we developed. It is based on the observed correlation between THM concentrations and TOC concentrations [21,22]. We estimated past concentrations THM*_pre-chlo_* to be the product of the current concentration (THM_2005–2011_) and the ratio between TOC concentrations in raw (TOC_RW_) and treated water (TOC_TW_):

(2) $$\frac{{\mathrm{THM}}_{pre-chlo}}{{\mathrm{THM}}_{2005-2011}}=\frac{{\mathrm{TOC}}_{RW}}{{\mathrm{TOC}}_{TW}}\;\;\to \;\;{\mathrm{THM}}_{pre-chlo}={\mathrm{THM}}_{2005‒2011}\times \frac{{\mathrm{TOC}}_{RW}}{{\mathrm{TOC}}_{TW}}$$

Method 2 assumes:

(1) The constancy of the organic matter content of raw water;
(2) The constancy of the ratio of the THM concentration at the outlet of the DWTP to the TOC concentration at the chlorine injection point, irrespective of the location of the chlorine injection point. This implies that:
  (a) The qualitative composition of organic matter responsible for THM formation did not differ between raw and treated water, despite the huge reduction seen in the amount of organic matter.
  (b) Chlorine was not a limiting factor of THM formation, either in pre- or post-chlorination. We assumed that raw water quality remained stable from 1960 onward and that TOC at the chlorine injection point determined the total amount of THM produced.

The average THM concentration for each DWTP from 1960 to 2000 (THM_1960–2000_) was calculated by using the average of THM*_pre-chlo_* and THM_2005–2011_, weighted by the number of years with and without pre-chlorination. As data for year t, when pre-chlorination was banned for a given DWTP, were not available for all DWTP, t was randomly chosen for each DWTP following a uniform distribution between 1980 and 2000:

(3) $${\mathrm{THM}}_{1960-2000}=\left({\mathrm{THM}}_{pre-chlo}\times \left(t-1960\right)\right)+\left({\mathrm{THM}}_{2005-2011}\times \left(2000-t\right)\right)$$

#### 2.4.2. Estimating Past THM Levels in Treated Groundwater

Whether chlorination occurred before 2003 or not was determined by the presence or not of measurements of free chlorine in the SISE-Eaux database from 2000 to 2003. We assumed that past chlorination practices were similar to current ones and that the content of organic matter of raw water did not significantly change over the study period, and therefore that THM_1960–2000_ = THM_2005–2011_. If no residual chlorine measurements were available, we assumed that the water was not chlorinated (i.e., neither pre- nor post-) before 2003 (THM_1960–2000_ = 0).

### 2.5. Calculating National and District Averages

Once the estimating concentrations of THMs for 1960–2000 and 2005–2011 were determined for each DWTP, we calculated district and national averages weighted by the size of the population.

## 3. Results

**Figure 1:**
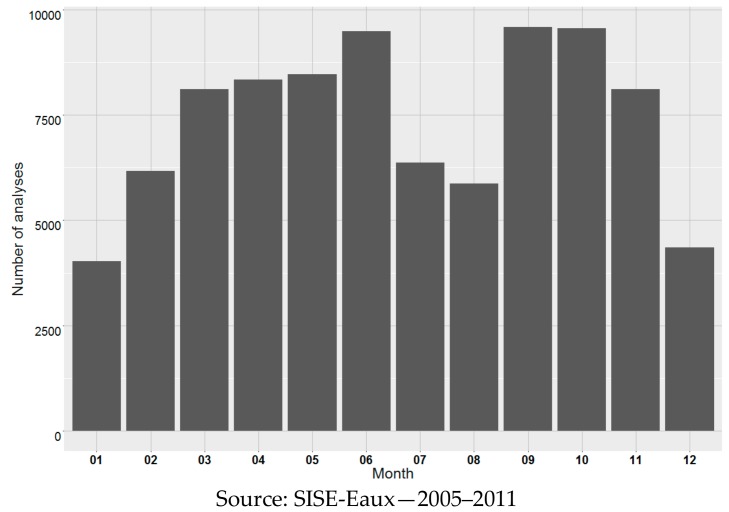
Number of samples for the measurement of THM, per month (13,732 DWTP). THM: trihalomethanes.

The sampling plan, elaborated at the district level, shew seasonal variations in sampling frequency with high density in spring and fall and lower density in summer and winter (Figure 1).

**Figure 2:**
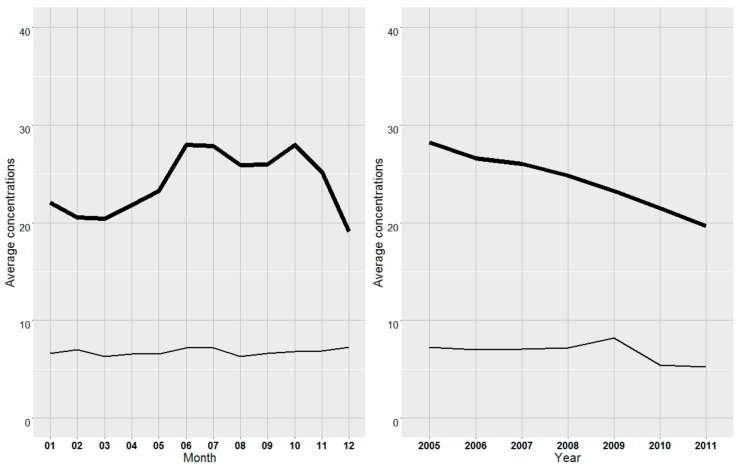
Average THM concentrations of (μg·L^−1^), by month and year depending on the nature of the water (line in bold: surface water; thin line: groundwater).

The average current THM concentrations in France (11.7 g·L^−1^) depend on the nature of the resource (10.3 for groundwater origin vs. 20.3 µg·L^−1^ for surface water origin) in direct relation with their content in organic matter). TOC in raw surface water ranged 0.2–15.0 mg·L^−1^ (median = 1.8 average = 2.7) and TOC in corresponding finished water ranged 0.2–5.7 mg·L^−1^ (median = 1.2 average = 1.3). THM concentrations in water of surface origin peaked in summer (Figure 2) and linearly decreased during the period 2005–2011(Figure 2), since THM concentration in finished groundwater remained quite stable across seasons and years.

With regard to past exposure, Method 1 and Method 2 yielded similar average THM concentration estimates in France (17.3 and 17.5 µg·L^−1^, respectively), reflecting a dramatic decrease in comparison with current exposure (Table 1).

The two methods used to estimate past contamination yielded similar average contamination estimates. Method 2 saw a slightly lower prevalence of low concentrations (35% vs. 36%) and a slightly higher estimate of high concentrations (8% vs. 7%). (Table 2).

THM concentrations decreased by one third between 1960–2000 and 2005–2011. This decrease was driven by a fall in concentrations in the most contaminated districts. One percent of the population was exposed to concentrations over 50 µg·L^−1^ in 2005–2011, compared with 8% in 1960–2000. This can be attributed to treatment progress: the banning of pre-chlorination and further removal of organic matter prior to post-chlorination.

For groundwater, a slight increase in average concentrations of THM between 1960–2000 and 2005–2011 was observed (Table 2), linked to the roll-out of post-chlorination.

**Figure 3:**
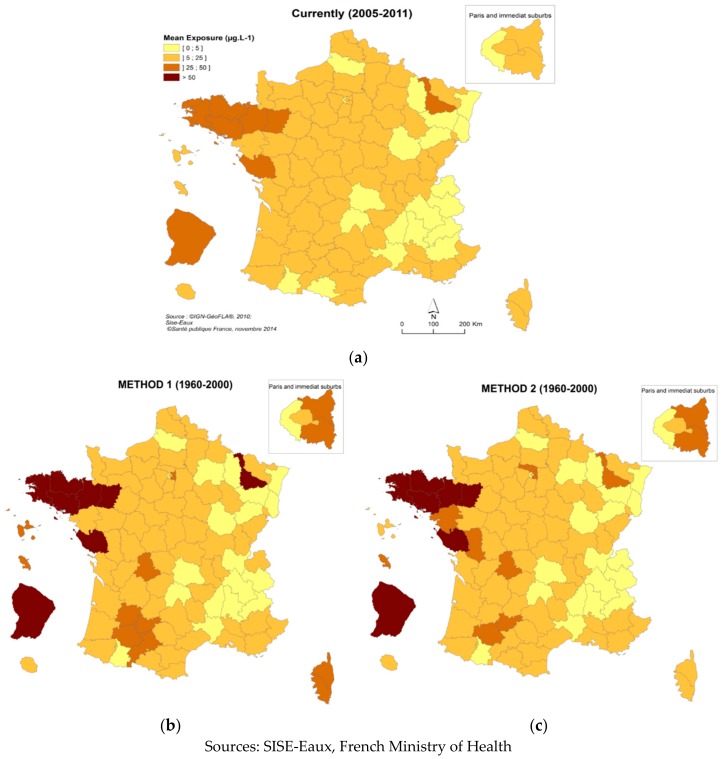
Average THM concentrations in the 100 French districts. (**a**) Current concentrations (2005–2011); (**b**) Past concentrations (1960–2000, Method 1); (**c**) Past concentrations (1960–2000, Method 2).

Large variations in exposure were observed between France’s 100 (at the time of the study) administrative districts, these spatial variations broadly remaining unchanged throughout the study period (Figure 3). The districts with the highest THM concentrations were in western France (Vendée with 38.6 µg·L^−1^; Morbihan and Ille-et-Vilaine with 34.5 µg·L^−1^), in North-Eastern France, (Meurthe-et-Moselle with 28.2 µg·L^−1^), and overseas (Guyane with 31.1 µg.L^−1^; and Martinique with 22.3 µg·L^−1^). This disparity essentially reflects the proportion of the population supplied with surface water. Current THM contaminations may thus be worse than past contamination in districts where groundwater is mainly used as a drinking water resource, because of the implementation of chlorination between the two periods considered. Indeed, among the 31 districts where the THM concentration increased, all except one were mainly fed by groundwater (>75% of the distributed flow). Within this category, the difference between past and current THM average concentrations ranged from 0 to 7.7 µg·L^−1^, with a mean of 0.9 µg·L^−1^ and a median of 0.4 µg·L^−1^. In contrast, among the districts where water quality improved (*N* = 67), the difference ranged from 0 to 40.7 µg·L^−1^, with a mean of 9.0 µg·L^−1^ and a median of 6.8 µg·L^−1^, the greatest improvements being associated with more than 50% of the production provided by surface water.

## 4. Discussion

### 4.1. Added Value of Method 2

THM concentrations gathered between 2005 and 2011 provided estimates for current exposure to CBP. In France, current concentrations should not be used as an estimate of past exposure since important changes in the treatment processes driving THM concentrations have occurred in the last few decades. However, these estimates can be useful for predicting the future impact and testing the potential effects of management actions, such as lowering the regulatory limit value for THM concentrations in tap water.

To reconstruct the past exposure of the French population to CBP, we used two methods that explicitly accounted for changes in water treatment methods. This helped estimate exposure over a long period in the absence of past THM measurements. In particular, these methods took the end of pre-chlorination of surface water into account. This element is critical, because ending pre-chlorination resulted in a 50% decrease in CBP exposure. The first method consisted in applying an overall increase coefficient to current exposure, as a proxy of past exposure associated with the presence of pre-chlorination. However, this method did not account for actual levels of organic matter in the surface resources nor for the capacity of the treatment to reduce TOC concentration in water. We therefore developed a second method which, like the first, relied on the reconstruction of the history of changes in treatment, but importantly accounted for local specificities, i.e., the content of organic matter in local resources measured by evaluating the TOC in raw water and the capacity of local treatment to reduce the TOC. This was measured using the ratios of TOC in raw and finished water. TOC data are available in the SISE-Eaux database from 2000 onward for all DWTP that use surface water. Method 2 assumed that TOC data measured at the chlorine injection point roughly determine the amount of THM formed. Some data and studies support this hypothesis [21]. The fact that Method 2 yielded a national level estimate for exposure similar to the one obtained through pilot simulation (Method 1) is in favors of its validity at a national level. Furthermore, Method 2 provided a more contrasted and realistic distribution of exposure than Method 1, due to the spread of the DWTP TOC_RW_/TOC_TW_, the 25th and 75th percentiles of which being, respectively, 1.06 and 2.55.

Estimates of past THM levels were reassessed in the USA, for a case-control epidemiologic study conducted in Iowa [23]. In the original analysis, surface water treatment plants were assigned one of two possible THM levels depending on the point of chlorination. The reassessment considered multiple treatment/disinfection scenarios and water quality parameters with actual DBP measurements, in order to develop estimates of past levels. As in Method 2, the water quality and treatments impacting DBP formation were taken into account. We therefore recommend Method 2 for estimating past exposure in France, in the absence of THM data.

### 4.2. Uncertainties Due to Data

The main sources of uncertainty affecting estimates of past THM contamination levels in distributed water stem from the hypothesis made on the model of reconstruction of past exposure. This hypothesis cannot be validated given the absence of THM data for DWTP that included pre-chlorination, and the gaps and uncertainties in the data detailed below.

#### 4.2.1. THM Limits of Quantification

Thirty seven percent of THM analyses had at least one compound whose concentration was below the limit of quantification and were thus categorized < LQ (Limits of Quantification). We observed large gaps between low and high exposure estimates for the least contaminated DWTP. The effect of these censored data was taken into account in the confidence intervals and exposure estimates.

#### 4.2.2. Point of Collection of Water Samples for THM Analysis

We estimated exposure as the mean THM concentration at the outlet of the DWTP. With current chlorination practices, THM concentrations increase during water transit in distribution networks. Within a single WDZ, the THM concentration can increase by a mean factor of 2 to 6 from the outlet of DWTP to consumers’ taps [24], and this partly depends on the time for water transit. Consequently drinking water THM is greatly underestimated, especially in widespread networks and networks supplied with water containing high levels of organic matter, i.e., mainly networks supplied with surface water, where re-chlorination stations maintain a chlorine residual everywhere within the distribution network. Thus, the greatest increase in THM concentration from DWTP to taps is seen in rural districts served by a few, spread out WDZs fed by surface water (e.g., in the Loire-Atlantique district), while the lowest increases are seen where populations are dense and distribution networks compact.

#### 4.2.3. Uncertainty about Treatment Evolution

Due to the lack of easy-to-obtain information about the dates of implementation and of cessation of pre-chlorination at the DWTP level, we assumed that all DWTP treating surface water used pre-chlorination from 1960 to 1980. We randomly generated the date of cessation within the next 20-year period of time (i.e., 1980–2000). Several simulated dates resulted in variations under 3% between the estimates and the average past exposure of the French population supplied with surface water. This relative insensitivity of exposure to simulated dates of pre-chlorination cessation at the national level does not imply insensitivity at the district level, given the smaller number of DWTP involved. Maximum uncertainty was found for districts supplied by a small number of WDZ (e.g., in the Loire-Atlantique district, supplied by only 9 WDZ).

In addition to the uncertainty about the period when pre-chlorination prevailed, we identified two categories of water systems for which high uncertainty hindered the assessment of past THM concentrations. The use of a common model for all WDZ fed by groundwater is questionable in the case of groundwater influenced by surface water. This category covers karstic and shallow alluvial aquifers. In the case of karstic water, clarification facilities may have been implemented during the time period considered for exposure to cope with turbidity spates. Since the vulnerability of aquifers to surface water influence is not clearly labelled in the SISE-Eaux database, no specific processing has been implemented in model 2. Ozonation also may change the quantity and the quality of the residual organic matter. The presence of ozonation could reduce the risk of bladder cancer associated with THM [12]. Again, among the WDZ fed by surface water, we cannot distinguish in the SISE-Eaux database those whose treatment includes ozonation from those that do not. Accordingly, it would be worth it to collect additional information on treatment evolution within the sectors included in an epidemiological study, in particular the actual date when prechlorination stopped, the presence of ozonation, and the implementation of clarification facilities for WDZ fed by karst groundwater.

#### 4.2.4. Existence of Multiple Water Resources

Twenty-five percent of WDZ and 42% of the French population are supplied by several DWTPs. Twelve percent of the population is supplied by more than three DWTPs. However, the contamination of one WDZ is estimated as the arithmetic average of the concentration for all DWTPs feeding into that WDZ, without weighting it by the volume of water supplied by the DWTP, creating a bias. This resulting bias is nevertheless moderated by the fact that the greater the volume supplied by the DWTP, the greater the frequency of regulatory sampling.

#### 4.2.5. Low Frequency of THM Analyses

The annual number of analyses required by regulations is low for DWTPs serving fewer than 500 inhabitants (between 0.1 and 0.5 analyses per year). The average THM concentrations over the period from 2005–2011 were estimated based on one to four analyses, leading to very imprecise mean contamination estimates, given the variations in concentrations over time. However, the corresponding population accounted for only 3% of the French population, almost all exclusively (98%) supplied with groundwater with very low levels of contamination, which remains quite consistent throughout the year. The bias that this introduced in average contamination estimates was therefore negligible at the district and national levels. The problem is more serious for DWTPs that supply water to between 500 and 4999 inhabitants. Even though a higher number of measurements was available for these DWTPs (one per year, seven over the studied period), the population supplied represented only 22% of the whole French population, 19% of whom being supplied only surface water. For DWTPs supplying water to over 5000 inhabitants, the number of samples (5–12 samples per year) provided an acceptable accuracy in average contamination assessment at the district level.

#### 4.2.6. Non-Random THM Sampling Strategy

Current French regulations establish the annual number of analyses that must be collected, without specifying how these analyses should be distributed throughout the year. Sampling strategies differ noticeably from one district to another, based on the choices of health authorities’ district representatives. Data show that some local authorities search for the maximum THM concentrations, leading to over-represented summer measurements, while others avoid conducting sampling campaigns during the summer. The average concentration estimates are subject to biases resulting from the combination of seasonal variations of concentrations and district sampling practices. We controlled for this potential bias in DWTPs fed by surface water, by correcting the measured concentrations for seasonal variations modelled at the district level.

#### 4.2.7. Conclusion: Uncertainties that Matter

THM data related to surface water resources were corrected for by using non-random THM sampling strategy. Inaccuracy due to censored analyses (<LQ) was incorporated into the confidence intervals. The main source of non-corrected uncertainty affecting estimates of past THM contamination in distributed water was the date when pre-chlorination of the DWTP ended. This resulted in possibly large errors in mean contamination estimates at the WDZ level, moderate errors at the district level, and a negligible error at the national level.

### 4.3. Ways Forward

#### 4.3.1. Water Quality Management

Results from Method 2 suggest that THM exposure halved between 1960–2000 and 2005–2011, in direct correlation with the end of pre-chlorination of surface water. This change in treatment firstly translated into a dramatic decrease in extreme exposure: from 7% to 1% for those exposed to more than 50 µg·L^−1^, and from 24% to 5% for those whose tap water came from surface water resources. The decrease in exposure continued over the 2005–2011 period despite reinforced disinfection procedures. This demonstrates the efforts of water operators to reduce soluble organic matter in water before chlorination [25] and adapt their strategy to maintain residual chlorine in the distribution network in a more parsimonious way (i.e., by injecting less chlorine at the outlet of the DWTP and counterbalancing this cut with appropriate re-chlorination points within the WDZ).

#### 4.3.2. From Exposure to Health Impact

Accurately estimating past exposure is a major limitation for retrospective epidemiological studies focusing on cancer and diseases with long incubation periods. We developed a new method to estimate past THM contamination of tap water across France. The present study’s greatest limitation is inaccuracy about exposure to THM in surface water-fed WDZs. This problem is the result of not knowing exactly when pre-chlorination for specific WDZs was abandoned. However, this inaccuracy decreases appreciably at the district level, due to the law of large numbers, and becomes negligible when assessing the national average exposure. Accordingly, this method is suitable for epidemiological studies and health impact assessments in France, depending on the geographical scale considered. More specifically, it is only a limitation for epidemiological studies requiring the estimation of exposure to the DWZ. For health impact assessments at a larger geographical scale, this limitation is greatly reduced.

Besides the limitation arising from not knowing when pre-chlorination was abandoned, our choice to consider THM samples at the outlet of the DWTP clearly led to a systematic underestimation of exposure levels. Having said that, most epidemiological studies [11,12,13] also consider these data for the same practical reasons which guided our choice: data availability and representativeness. However, when carrying out a health impact assessment, replicating the exposure assessment design formerly used in epidemiological studies selected to build the concentration-risk function, means that unbiased risk estimates can be made. In other words, unbiased risk estimates may be assessed from our biased exposure estimate, provided that the methods used for exposure assessment are homogenous between both the Health Impact Assessment and the studies selected to build the concentration-risk function formulation.

Estimating past exposure is a challenge faced by all epidemiological studies dealing with long exposure outcomes. Our study points out the crucial importance of considering the evolution of treatments in past exposure modeling. It could be adapted to other countries, for example, countries in the European Community, which share a common framework for water management and quality monitoring. The exposure of the population is however the result of a combination of the contamination of tap water and the user behavior. People are exposed more to THMs through showering and bathing than drinking tap water [14]. However, consumer behavior may have evolved during the time period considered for exposure assessment. In particular, the time given to bathing and showering has most likely increased from 1960 to nowadays, resulting in more exposure to constant contamination. Consequently, THM concentration at the outlet of DWTP may be considered an indicator of the contamination at the consumer’s tap but only a proxy of exposure, if the changes in exposure behaviors are ignored.

## 5. Conclusions

In this article, we assess the current and past concentrations of THMs from data collected in the framework of European regulations. We observed a dramatic decrease in tap water contamination by THMs in France, with an average concentration of 11.7 µg·L^−1^ in 2005–2011, vs. 17.3 µg·L^−1^ in 1960–2000. The population supplied with surface water was two times less potentially exposed in 2011 than in 1960, but still two times more exposed than the population supplied with groundwater. Geographical inequalities remained due to the type of resources operated.

Our study confirms the crucial importance of considering the evolution of treatments in the past exposure modeling. It proposes an alternative approach to measuring past exposure of the French population to THMs, based not only on historical treatment method changes impacting THM formation, something previously done elsewhere, but also on the organic content in water resources. The model can be used for health impact assessment at the country and district levels. At the WDZ level, our estimates were inaccurate due to a lack of knowledge about local treatment changes. This is the main way forward to improve the specificity of model 2 for an epidemiological use. 

## Figures and Tables

**Table 1:** Average concentrations of THM in finished water (2005–2011), France.

THM (in µg·L^−1^)	Sources		
	Groundwater	Surface Water	Total
	Mean (min; max)	Mean (min; max)	Mean (min; max)
2005–2011	10.3 (8.6; 11.9)	20.3 (18.9; 21.6)	11.7 (10.3; 13.2)
1960–2000 (Method 1)	8.9 (7.5; 10.2)	43.8 (41.0; 46.7)	17.5 (15.8; 19.2)
1960–2000 (Method 2)		39.0 (36.6; 41.4)	17.3 (15.7; 18.9)

Sources: SISE-Eaux, French Ministry of Health.

**Table 2:** Distribution of the French population in terms of current and past exposure to THM (in %).

THM (µg·L−1)	Ground Water				Surface Water				Total			
	0–5	5–25	25–50	>50	0–5	5–25	25–50	>50	0–5	5–25	25–50	>50
2005–2011	29%	66%	5%	0%	8%	42%	45%	5%	32%	58%	9%	1%
1960–2000 (Method 1)	36%	59%	5%	0%	4%	21%	46%	28%	35%	43%	15%	8%
1960–2000 (Method 2)					6%	26%	44%	24%	36%	44%	13%	7%

Sources: SISE-Eaux, French Ministry of Health.
